# Genotype/Phenotype Relationship in a Consanguineal Family With Brugada Syndrome Harboring the R1632C Missense Variant in the *SCN5A* Gene

**DOI:** 10.3389/fphys.2019.00666

**Published:** 2019-05-28

**Authors:** Michelle M. Monasky, Emanuele Micaglio, Giuseppe Ciconte, Sara Benedetti, Chiara Di Resta, Gabriele Vicedomini, Valeria Borrelli, Andrea Ghiroldi, Marco Piccoli, Luigi Anastasia, Vincenzo Santinelli, Maurizio Ferrari, Carlo Pappone

**Affiliations:** ^1^ Arrhythmology Department, IRCCS Policlinico San Donato, San Donato Milanese, Milan, Italy; ^2^ Laboratory of Clinical Molecular Biology and Cytogenetics, IRCCS San Raffaele Hospital, Milan, Italy; ^3^ Genomic Unit for the Diagnosis of Human Pathologies, Division of Genetics and Cellular Biology, IRCCS San Raffaele Hospital, Milan, Italy; ^4^ Vita-Salute San Raffaele University, Milan, Italy; ^5^ Stem Cells for Tissue Engineering Laboratory, IRCCS Policlinico San Donato, San Donato Milanese, Milan, Italy; ^6^ Department of Biomedical Sciences for Health, University of Milan, Milan, Italy

**Keywords:** Brugada syndrome, sudden cardiac death, genetic testing, arrhythmia, *SCN5A*, sodium channel, cardiomyopathy, variant

## Abstract

Brugada syndrome (BrS) is a known cause of sudden cardiac death. The genetic basis of BrS is not well understood, and no one single gene is linked to even a majority of BrS cases. However, mutations in the gene *SCN5A* are the most common, although the high amount of phenotypic variability prevents a clear correlation between genotype and phenotype. Research techniques are limited, as most BrS cases still remain without a genetic diagnosis, thus impairing the implementation of experimental models representative of a general pathogenetic mechanism. In the present study, we report the largest family to-date with the segregation of the heterozygous variant NM_198056:c.4894C>T (p.Arg1632Cys) in the *SCN5A* gene. The genotype-phenotype relationship observed suggests a likely pathogenic effect of this variant. Functional studies to better understand the molecular effects of this variant are warranted.

## Background

The Brugada syndrome (BrS) is diagnosed by the presence of a coved-type ST-segment elevation (type 1 BrS pattern) in the right precordial leads on the electrocardiogram (ECG) that occurs either spontaneously or after administration of a sodium channel blocking agent, such as ajmaline (Antzelevitch et al., 2016), which reveals the type 1 pattern. Patients with this pattern are at increased risk of sudden cardiac death (SCD) (Antzelevitch et al., 2016). The arrhythmias are caused by the presence of an arrhythmogenic substrate (AS) usually found in the epicardial surface of the right ventricle (RV) (Nademanee et al., 2011; Zhang et al., 2015), which can be eliminated by trans catheter radiofrequency (RF) ablation. Ajmaline can be used to fully visualize the extent of the AS and improve the success of the procedure (Pappone et al., 2017).

The true prevalence of BrS is currently unknown, as SCD may be the first clinical manifestation in affected individuals. Many patients display a wide degree of clinical variability, even among the same family. BrS is considered as an inherited autosomal dominant disease with incomplete penetrance (Chen et al., 1998; Nademanee et al., 2011; Lieve and Wilde, 2015). Variants in the *SCN5A* gene are found in about 15–30% of BrS cases (Kapplinger et al., 2010). *SCN5A* mutations are found in a number of pathologies, such as arrhythmogenic right ventricular cardiomyopathy, atrial standstill type 1, atrial fibrillation, left ventricular noncompaction, dilated cardiomyopathy, long QT syndrome type 3, sick sinus syndrome type 2, idiopathic ventricular fibrillation, and heart block type 1A (Gosselin-Badaroudine et al., 2014; Zaklyazminskaya and Dzemeshkevich, 2016; Monasky et al., 2018), making it difficult to predict a phenotype from novel variants within *SCN5A*. In the case of BrS, *SCN5A* variants are associated with a loss of function of the voltage-gated sodium channel subunit (Na_V_1.5) (Di Resta et al., 2015; Sieira et al., 2016; Curcio et al., 2017).

Although a significant enrichment in rare coding variations in patients versus controls was observed only for the *SCN5A* gene by burden test (Le Scouarnec et al., 2015), genetic mutations are still not detected in the majority of BrS patients (Sonoda et al., 2018), making BrS genetically elusive in many cases. In addition, the importance of genetic background has been highlighted in different studies, questioning Mendelian inheritance and proposing more complex oligogenic models (Bezzina et al., 2013, 2015; Di Resta et al., 2015). Better understanding of the genetics of BrS could assist clinicians in patient risk assessment, especially because ajmaline tests might have a low, but present, false negative rate (Therasse et al., 2017). However, research studies are limited by the lack of understanding of the human genetics in BrS. In the present study, we report a family with a heterozygous variant NM_198056:c.4894C>T (p.Arg1632Cys) in the *SCN5A* gene. This variant has only been described twice before, once in a single patient with a complex arrhythmogenic phenotype (Nakajima et al., 2015) and once in two siblings with vastly different phenotypes (Garcia-Molina et al., 2016). Thus, the phenotype associated with the variant described herein is uncertain in the current literature. In the present study, we report for the first time the genotype-phenotype correlation in several individuals, providing the strongest evidence to-date as to the possible effects of this variant.

## Case Presentations

**Figure 1:**
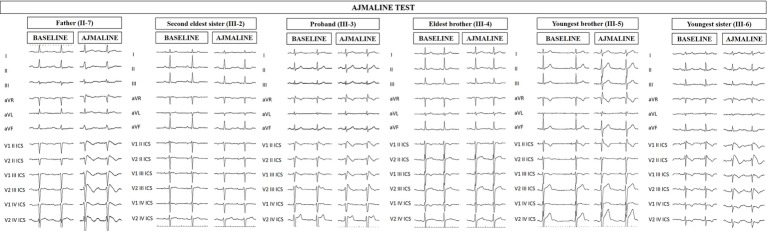
Electrocardiograms of proband and family members at baseline and after ajmaline challenge. Proband’s father (II-7), proband (III-3), and proband’s youngest sister (III-6) exhibit the BrS type 1 pattern after ajmaline administration, confirming the BrS diagnosis. Proband’s second eldest sister (III-2) and brothers (III-4 and III-5) are ajmaline negative.

**Figure 2:**
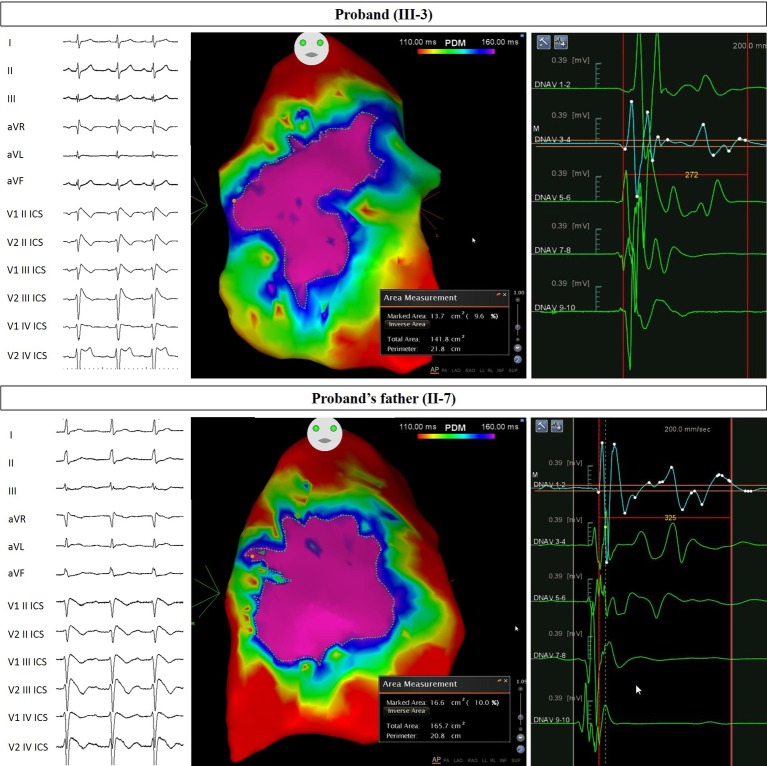
Arrhythmogenic substrate characterization in Proband and Father. Electrocardiogram (left) demonstrates the type 1 BrS pattern after ajmaline administration, confirming the diagnosis. Potential duration map after ajmaline infusion (center) reveals the full extent of the arrhythmogenic substrate. The area of the substrate exhibiting potential durations at least 160 ms in duration is denoted by the “marked area” and is 13.7 cm^2^ for Proband and 16.6 cm^2^ for Proband’s father. The duration of fragmented potentials (right) were prolonged and measured 272 ms for Proband and 325 ms for Proband’s father.

The proband is a 32-year-old male of Italian origin with a history of syncope and hypothyroidism treated with substitutive levothyroxine. He performed an arrhythmogenic evaluation after a routine ECG (performed for known hypothyroidism) revealed a type 1 BrS pattern (Figure 1). Thus, the proband underwent electrophysiological study (EPS), which was negative. The patient received ajmaline to reveal the full extent of the AS prior to substrate catheter ablation (Figure 2).

### Genetic Studies

Genetic testing was performed on peripheral blood-extracted genomic DNA with massive parallel sequencing (NGS) using Illumina TruSight Cardio enrichment and MiSeq platform. NGS yielded a coverage >20X in 99.6% targeted regions and an average depth = 279X. Variants were called exploiting both commercial and in-house pipelines based one BWA, Smith-Waterman Algorithm, Freebayes, SnpSift-SnpEFF, MiSeq reporter. Sixteen BrS genes were analyzed: *CACNA1C, CACNA2D1, CACNB2, GPD1L, HCN4, KCND2, KCND3, MOG1, PKP2, RANGRF, SCN10A, SCN1B, SCN2B, SCN3B, SCN5A*, and *TRPM4*. Variants with a minor allele frequency > 0.01 according to ExAC database and synonymous or intronic variants if not reported as pathogenic were filtered out.

**Figure 3:**
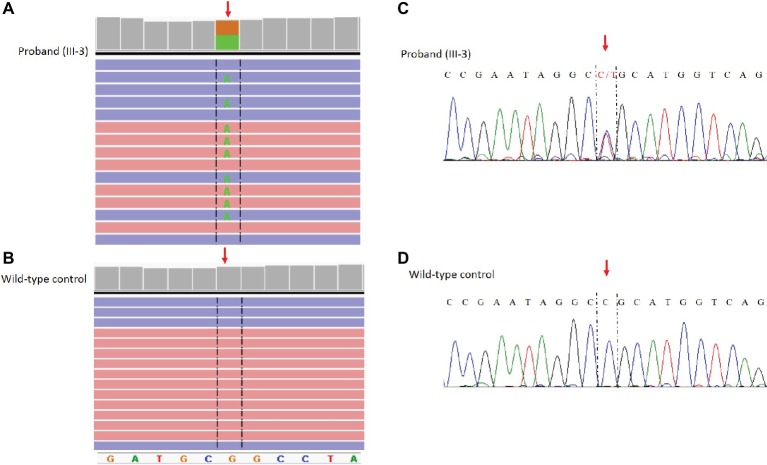
Identification of the c. 4894C>T (p.Arg1632CyS) missense mutation in the *SCN5A* gene. NGS paired-end reads loaded in the IGV genome browser. The arrow indicates the position of the single nucleotide variation in *SCN5A* gene in the proband **(A)** compared to a wild type control sample **(B)**. *SCN5A* gene is in the reverse orientation on the chromosome. Sanger sequencing electropherogram confirm the presence of the variants in the proband **(C)** and the absence in a wild type control **(D)**.

The NM_198056:c.4894C>T (p.Arg1632Cys) variant identified by NGS in the *SCN5A* gene was confirmed by Sanger sequencing using standard protocols (Kieleczawa, 2005) and the following primers: Forward_GCACAGTGATGCTGGCTGGAA Reverse_GCAGAGTGGGGTCGCAGTAGG (Figure 3).

### Assessment of Family Members

**Figure 4:**
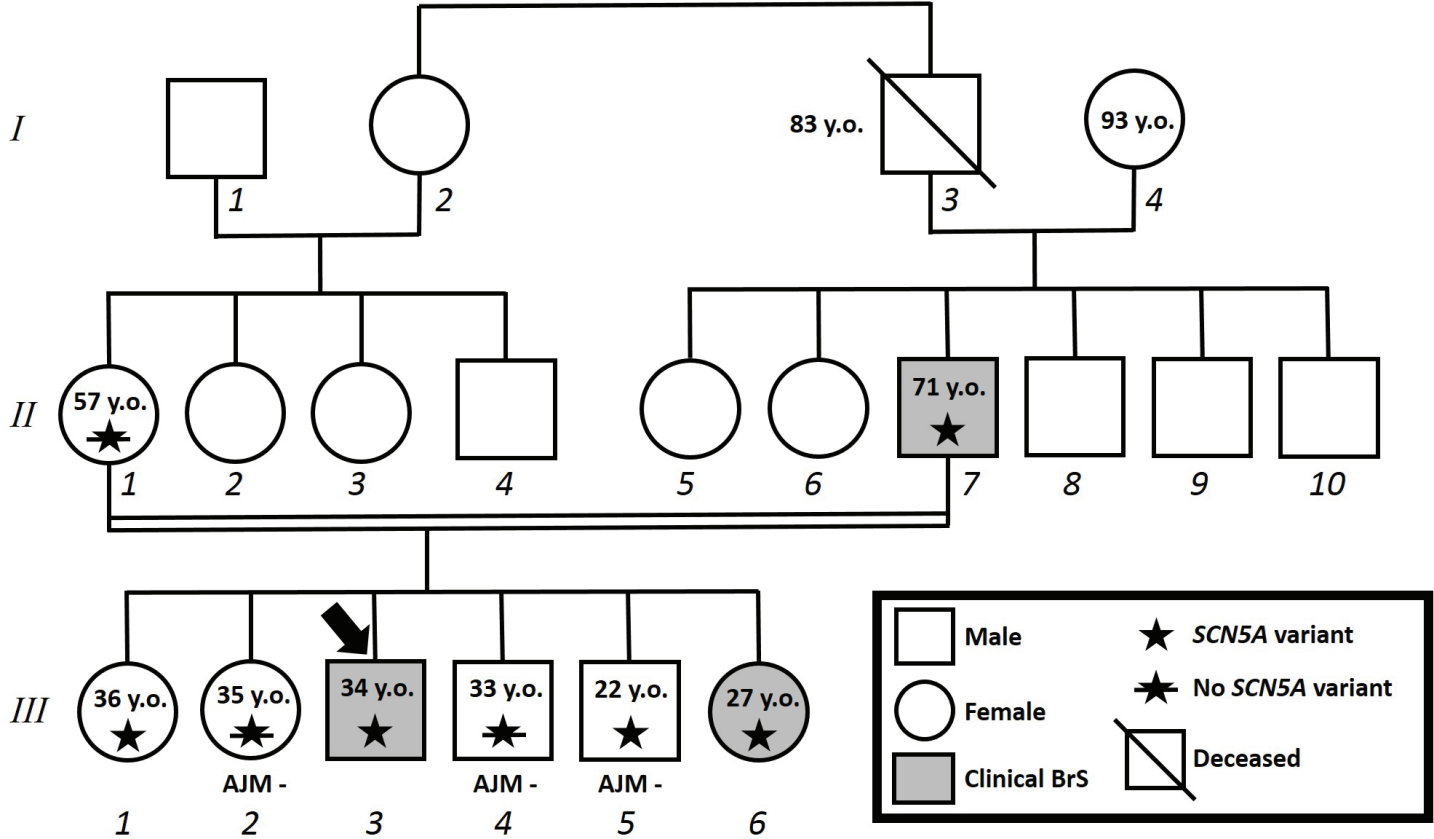
Family pedigree. Proband identified with arrow. Square: male; circle: female; slash: deceased; grayed: clinically affected by Brugada syndrome; star: molecularly confirmed *SCN5A* variant; star with slash: genetic test for *SCN5A* variant performed but negative.

The proband’s father is a 64-year-old male diagnosed at the age of 55 with arterial hypertension (pharmacologically treated with ACE inhibitors, successfully) and binodal heart disease. He came to our attention for familial history of BrS (in his son, the proband: see family tree, Figure 4). He received arrhythmologic evaluation by his family doctor, who recommended ajmaline challenge (Figure 1, Table 1) and an EPS. The results of both of these exams were positive, so an implantable cardioverter device was implanted. After a couple of months, he underwent both binodal disease and BrS arrhythmogenic substrate ablation with a technique based on a transcatheterial application of radiofrequency (Figure 2). Both these procedures were successful and without any operative or postoperative complications. It is interesting that after the two ablations, the arterial hypertension improved, requiring a lesser dosage of antihypertensive drugs that he had taken for the previous 8 years. He underwent genetic counseling and was advised to undergo genetic testing for BrS. This test was performed on peripheral blood-extracted genomic DNA and sought only the heterozygous variant NM_198056:c.4894C>T (p.Arg1632Cys) in the *SCN5A* gene. This test was positive, confirming a paternal origin for the variant discovered in the proband. The mother had also been genetically tested, due to the consanguinity with her husband, and she was negative for the variant found in her son.

**Table 1:** Electrocardiogram parameters at baseline and after ajmaline administration, results of electrophysiological study and genetic testing.

	Baseline				Ajmaline				EPS	*SCN5A* variant
	HR (bpm)	PR (ms)	QRS (ms)	QTc (ms)	HR (bpm)	PR (ms)	QRS (ms)	QTc (ms)		
II-7	68	290	95	410	72	365	138	510	Positive	Yes
III-2	63	186	85	365	69	234	111	401	Negative	No
III-3	88	220	110	420	–	–	–	–	Negative	Yes
III-4	67	170	107	402	85	222	120	450	Negative	No
III-5	44	181	106	390	59	250	125	445	Negative	Yes
III-6	69	244	103	430	75	330	122	485	Negative	Yes

Proband’s eldest sister (III-1) is 36 years old and asymptomatic. Her medical history is unremarkable and negative for arterial hypertension and binodal disease. However, she was referred for both ajmaline challenge and EPS due to a family history of BrS. She refused both of these procedures but accepted to undergo genetic testing to seek the heterozygous variant NM_198056:c.4894C>T (p.Arg1632Cys) in the *SCN5A* gene. The result was positive.

Proband’s second eldest sister (III-2) is a 35-year-old asymptomatic individual with an unremarkable medical history. The clinical arrhythmological evaluation was negative for arterial hypertension and binodal disease. However, she, like her sister, was referred for both ajmaline challenge and EPS due to family history of BrS. Both of these tests were negative (Figure 1). She also underwent genetic testing to seek the heterozygous variant NM_198056:c.4894C>T (p.Arg1632Cys) in the *SCN5A* gene, which was negative.

Proband’s eldest brother (III-4) is 33 years old and has an unremarkable medical history. The clinical arrhythmological evaluation was negative for both arterial hypertension and binodal disease. However, he, like his siblings, was referred for both ajmaline challenge and EPS due to family history of BrS. Both of these tests were negative (Figure 1), as was a genetic test for the *SCN5A* variant found in his family members.

Proband’s youngest brother (III-5) is a 22-year-old male affected by allergic asthma, vitiligo, and insulin-dependent diabetes with onset in a pediatric age. No antibodies dosage was available at the time of genetic counseling, but the association with vitiligo suggests an autoimmune pathogenesis (Jin et al., 2007). The patients suffered from recurrent lipothimic episodes, initially thought to be related only to type 1 diabetes. A 12-lead ECG performed because of family history raised the suspicion of BrS. The patient was then referred for EPS, ajmaline challenge, and genetic counseling. The EPS and ajmaline challenge were both negative (Figure 1). Genetic testing seeking the familial variant confirmed this patient harbors the paternally inherited heterozygous variant NM_198056:c.4894C>T (p.Arg1632Cys) in the *SCN5A* gene. This patient has not performed any genetic testing to assess whether his diabetes could be considered syndromic or not.

Proband’s youngest sister (III-6) is 28 years old with a medical history of autoimmune urticaria, autoimmune thyroiditis, and low blood pressure since a pediatric age. She experienced both lypothymic and syncopal episodes during fever and sometimes without fever as well. The arrhythmogenic evaluation performed because of her family history was negative for evidence of major arrhythmias or binodal disease. Considering her father’s condition, a 12-lead ECG was performed, which raised the suspicion of BrS. Therefore, an ajmaline challenge was performed and confirmed the BrS diagnosis (Figure 1). An EPS was performed, but the patient was not inducible. This patient was then found to be positive for the same variant in *SCN5A* that was discovered in her father.

### *In Silico* Predictions and Variant Classification

Several prediction tools were used to clarify the significance of the *SCN5A* variant: VarSome genetic database, SIFT, Polyphen2, Mutation Taster, AlignGVGD, and Provean. All of these softwares supported a damaging effect for the p.Arg1632Cys substitution. The Genomic Evolutionary Rate Profiling (GERP) value was noted, which is defined by VarSome as “a conservation score calculated by quantifying substitution deficits across multiple alignments of orthologues using the genomes of 35 mammals. It ranges from −12.3 to 6.17, with 6.17 being the most conserved” (Cooper et al., 2005). The GERP score for the *SCN5A* variant described herein was 4.5399. According to ClinVar (2018), no families in which the c.4894C>T heterozygous variant in the *SCN5A* gene segregates are provided to date (last update February 18, 2018). The allele frequency is 1: 113,644 in the general population (2019).

The c.4894C>T variant was classified as likely pathogenic according to ACMG criteria (Richards et al., 2015):

- PM1: Located in a mutational hot spot and/or critical and well-established functional domain (in this case S4 voltage sensor in domain IV)
- PM2: Extremely low frequency in general population (1/113,644 according to gnomAD) (gnomAD, 2019).
- PM5: Novel missense change at an amino acid residue where a different missense change determined to be pathogenic has been seen before: the p.R1632H was previously reported and determined to affect channel function by functional studies (Benson et al., 2003; Gui et al., 2010).
- PP3: Multiple lines of computational evidence support a deleterious effect on the gene or gene product
- PP1: Cosegregation with disease in multiple affected family members in a gene definitively known to cause the disease

## Discussion

In the present study, we report the genotype-phenotype correlation in the largest family to-date with this heterozygous variant in the *SCN5A* gene. This study not only underlines the importance of genetic testing to identify family members who require preventive interventions, highlighting its clinical importance.

Since sudden cardiac death may be the first symptom with which a BrS patient presents, preventive interventions are instrumental in saving these lives. Genetic testing is a noninvasive, and thus safe and painless, method for family member risk stratification. Thus, understanding better the genetics of BrS not only can help create better disease models but it also has a significant clinical impact.

The *SCN5A* variant described herein is currently, as of the time of this writing, of unknown significance in the commonly relied upon database VarSome (VarSome, 2018). *In silico* studies predicted this variant to be damaging, because it is located in a voltage sensitive domain (VSD): a region crucial for the correct function of the Na_V_1.5 protein (Wang et al., 2016). The activity of the Na_V_1.5 channel protein is controlled by a VSD (Noda et al., 1984) using the positive charges of both lysine and arginine residues. Mutations involving these amino acids have been demonstrated by other groups to reduce the steepness of voltage-dependent gating of the Na^+^ channel. Additionally, these particular kinds of mutations can generate gating pore currents different from the central ionic current, resulting in deep modifications of the Na_V_1.5 gating property (Sottas et al., 2013).

The clinical data in the present study support these *in silico* predictions and strengthen the hypothesis that this variant is likely pathogenic, as classified according to ACMG criteria (Richards et al., 2015) (see Case Presentations). Additionally, a recently published meta-analysis investigating the prognosis and risk stratification of *SCN5A* variants in BrS concluded that patients harboring an *SCN5A* variant were at higher risk of arrhythmic events (Yang et al., 2019), supporting our previous findings (Sommariva et al., 2013).

This variant has been previously described in a single patient who experienced a syncopal episode during exercise and who presented with atrial tachycardia, sinus node dysfunction, and BrS (Nakajima et al., 2015). Additionally, the same variant has been described in a brother and sister exhibiting vastly different phenotypes (Garcia-Molina et al., 2016). In that study, a negative T wave and right bundle branch block were observed in the brother, who also tested positive for BrS during a flecainide test. However, the sister was described as a “healthy carrier” who “exhibited a normal baseline and drug challenged ECG.” The authors concluded that there was “no evidence that this variant co-segregated with the disease.” It is important to note that the authors additionally described two additional variants in the brother, and that the reader is left to assume that the drug challenge performed in the sister was a flecainide test. However, flecainide tests are not as powerful as ajmaline tests in unmasking the type 1 BrS pattern and may result in false negative results (Wolpert et al., 2005).

It is significant to note for this study that the proband and his partner are consanguineous first cousins. Consanguinity increases the risk of inheriting pathogenic variants in both alleles of a disease gene, therefore leading to higher reproductive risk (Saleheen et al., 2017). It seems likely that consanguinity plays a role for arrhythmogenic conditions as well (Al-Hassnan et al., 2017). However, it must be additionally noted that, in spite of the consanguinity, proband’s mother tested negative for the familial variant. This finding increases the clinical significance of this variant for the disease that segregates in proband’s family. However, consanguinity may lead to the transmission of a modifier SNPs which, when inherited homozygously, may act like a genetic trigger for arrhythmias in symptomatic family members.

One family member (patient III-5) harboring the R1632C variant in the *SCN5A* gene tested negative during an ajmaline challenge. Type 1 diabetes in this patient may have influenced the outcome of the ajmaline test. Type 1 diabetes causes an insulin depletion that can influence both ST elevation and ST-T wave changes (Nishizaki et al., 2003). Therefore, the BrS clinical picture can change dramatically as a direct effect of variations in both glucose and insulin concentrations (Velazquez-Rodriguez et al., 2016). However, further studies are needed to better understand the relationship between BrS and diabetes.

## Concluding Remarks

This study provides the most convincing evidence to-date for the genotype/phenotype relationship for the NM_198056:c.4894C > T (p.Arg1632Cys) variant in the *SCN5A* gene. However, other mechanisms may complicate the genotype/phenotype correlation, including genetic background and particularly the presence of modifier polymorphisms. Further studies in larger groups of patients are therefore warranted to confirm these findings. Genetic testing is a valuable tool to identify at-risk family members and implement preventive interventions. Functional studies are warranted to better understand the molecular pathologies that result from this variant.

## Data Availability

The datasets generated for this study can be found in LOVD, https://databases.lovd.nl/shared/individuals/00222828.

## Ethics Statement

Written informed consent of human subjects included in this case series report was obtained for their participation in the study and for publication. The procedures employed were reviewed and approved by the local Ethics Committee.

## Author Contributions

MM, EM, GC, SB, CR, GV, VB, AG, MP, LA, VS, MF, and CP collected/analyzed the data. MM and EM wrote the manuscript. All interpreted results, critically reviewed/edited manuscript, and approved final version.

### Conflict of Interest Statement

The authors declare that the research was conducted in the absence of any commercial or financial relationships that could be construed as a potential conflict of interest.
